# Bladder perforation: an unusual complication caused by rectus sheath haematoma

**DOI:** 10.1093/jscr/rjab180

**Published:** 2021-05-27

**Authors:** Du Huynh Leo Phan, Anthony Stephen Leslie

**Affiliations:** Department of Surgery, Lismore Base Hospital, New South Wales 2480, Australia

## Abstract

Rectus sheath haematoma (RSH) is an uncommon cause of abdominal pain. Despite being previously viewed as a benign, self-limiting condition, there is increasing evidence suggesting significant local and systemic complications with RSH. We present a case of an 82-year-old female who developed a large RSH following prescription of therapeutic anticoagulation for her new onset atrial fibrillation. She subsequently developed significant haemodynamic collapse, which necessitated emergency radiological intervention. We describe a novel approach to prevent recurrence of bleeding by inserting a covered endovascular stent across the origin of inferior epigastric artery. We also describe a rare finding of bladder perforation, presumed secondary to pressure necrosis from the haematoma. Our report contributes to the growing evidence which suggests RSH, particularly secondary to anticoagulation in the elderly, can result in catastrophic complications. In addition, bladder perforation is a rare but possible complication that needs to be considered.

## INTRODUCTION

Rectus sheath haematoma (RSH) is an unusual cause of abdominal pain and frequently viewed as a benign, self-limiting condition [1]. However, there is growing evidence to suggest that RSH can result in significant life threatening complications. We present a case of RSH in an elderly patient that resulted in life threatening haemodynamic instability and bladder perforation, a rarely reported complication.

## CASE REPORT

**Figure 1:**
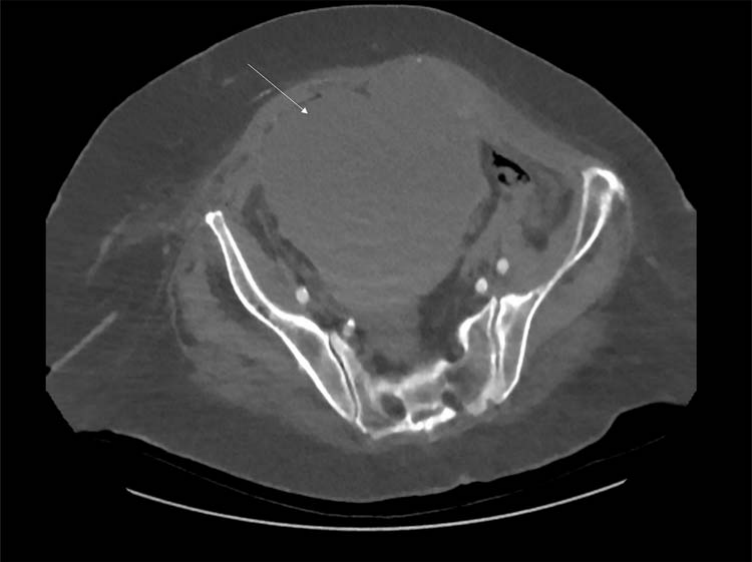
Large rectus sheath haematoma on computed tomography (white arrow).

A previously well 82-year-old female was referred for surgical opinion with worsening abdominal pain and loose bowel motions, after being anticoagulated (enoxaparin 90 mg twice daily) for 6 days under the medical team with new onset atrial fibrillation secondary to lower leg cellulitis. Relevant background includes hypertension and type two diabetes. Within 5 h of initial review, patient developed sudden haemodynamic collapse (systolic pressure 86 mm Hg, haemoglobin decreased from 114 g/L to 84 g/L and lactate raised to 13.5 mmol/L). Urgent computed topography showed a large right RSH measuring 151 × 137 × 128 mm extending into space of Retzius without contrast extravasation (Fig. 1). Due to concerns for active bleeding, an emergency radiological intervention by vascular team was arranged.

**Figure 2:**
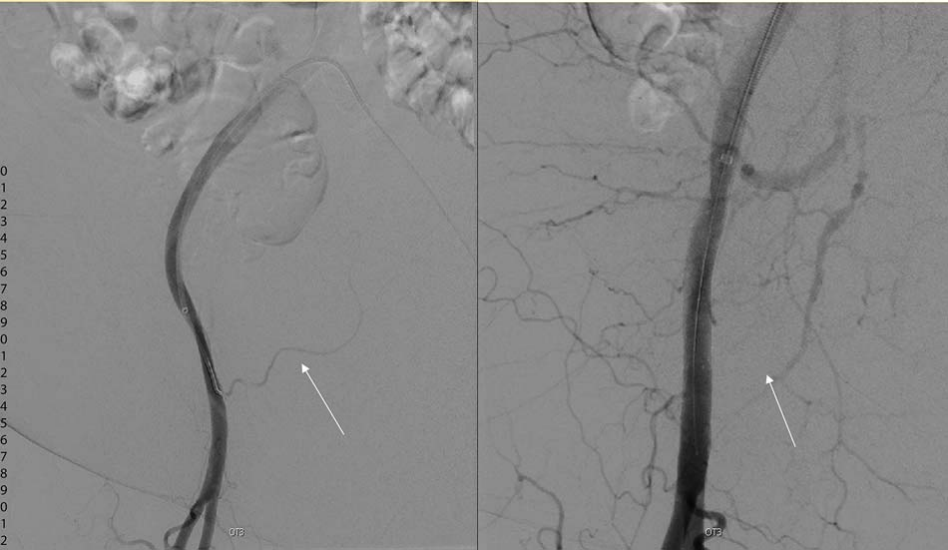
DSA showing left: flow through inferior epigastric artery (white arrow) and right: occlusion of inferior epigastric artery flow with covered stent in inferior epigastric artery.

During subtracted digital angiography (DSA), the right inferior epigastric artery (IEA) could not be cannulated due to a pre-existing atherosclerotic lesion but no active contrast extravasation was noted. An 8 × 50 mm, self-expanding covered stent was deployed in the external iliac artery (EIA) across the origin of the IEA to occlude inflow and prevent recurrence of bleeding (Fig. 2). The patient recovered haemodynamically and was transferred to intensive care unit (ICU). During this procedure, a cystogram performed via the indwelling Foley catheter confirmed the correct positioning and normal outlining of the bladder with no evidence of perforation.

**Figure 3:**
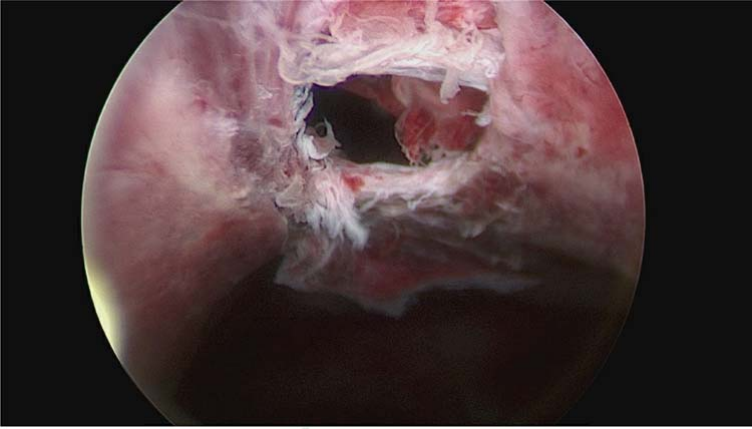
Cystoscopy showing perforation in anterior bladder wall communicating with the haematoma.

Day 1 post intervention, post-renal kidney injury due to mass effect of haematoma was suspected (creatinine 194 μmol/L and bilateral hydronephrosis on ultrasonographic study). Emergency cystoscopy (for consideration of ureteric stenting) revealed an anterior bladder wall perforation which communicated anteriorly with the haematoma in the extra-peritoneal space (Fig. 3). This was managed conservatively with long-term catheter and bilateral nephrostomy to decompress the dilated upper urinary tracts. Following 1 week admission in ICU, patient made gradual recovery with no evidence of re-bleeding following reintroduction of anticoagulation, nephrostomy tubes were removed 3 weeks post insertion uneventfully.

## DISCUSSION

RSH is an uncommon presentation, accounting for about 2% of abdominal pain causes [2]. RSH arises from rupture of either the inferior or superior epigastric arteries and therapeutic anticoagulation is an established risk factor [3]. Due to the absence of the posterior rectus sheath below arcuate line, RSH arising from the IEA may expand with less restriction and extend postero-inferiorly, as seen in our patient. Historically, RSH was managed conservatively without major complication. However, with the increasing usage of anticoagulation in the elderly, RSH with life threatening complication has been reported more frequently [1]. Radiological (contrast extravasation) or clinical evidence of active haemorrhage (haematoma expansion, haemodynamic compromise) warrants a consideration of active intervention. Although laparotomy or open ligation is often unnecessary, recent reports have described successful use of angiographic embolization using either gelfoam or gelatin sponge embolization to control bleeding [4, 5]. Our patient did not show active extravasation of contrast during DSA, however, a covered stent was deployed to occlude inflow from EIA and prevent bleeding recurrence. To our knowledge, this is the first report describing such an approach.

Complications of RSH are rare and may include infected haematoma, hypovolemic shock or intra-abdominal compartment syndrome [6]. We report an unusual complication of bladder perforation secondary to RHS. To our knowledge, only two previous reports have described similar finding in three other patients [7, 8]. Interestingly, all three were described to present with frank haematuria. In contrast, our patient did not have haematuria and perforation was only found during cystoscopy. We hypothesize that the rapidly expanding haematoma caused by the therapeutic enoxaparin resulted in pressure necrosis and perforation of the anterior bladder wall, which was stretched against the indwelling catheter. The absence of frank haematuria might be due to the active bleeding already controlled angiographically.

Our case contributes to the growing evidence that RHS can result in catastrophic haemodynamic collapse, particularly with the increasing use of anticoagulation in the elderly. In patients where selective embolization of the inferior epigastric vessel is not feasible, usage of covered stent to occlude inflow might be an option. Finally, bladder perforation is a rare but possible complication that needs to be considered.

## CONFLICT OF INTEREST STATEMENT

None declared.

## FUNDING

None.
